# GeneMANIA Prediction Server 2013 Update

**DOI:** 10.1093/nar/gkt533

**Published:** 2013-06-19

**Authors:** Khalid Zuberi, Max Franz, Harold Rodriguez, Jason Montojo, Christian Tannus Lopes, Gary D. Bader, Quaid Morris

**Affiliations:** ^1^The Donnelly Centre, University of Toronto, Ontario, Canada, ^2^Department of Computer Science, University of Toronto, Ontario, Canada, ^3^Department of Molecular Genetics, University of Toronto, Ontario, Canada and ^4^Department of Electrical and Computer Engineering, University of Toronto, Ontario, Canada

## Abstract

GeneMANIA (http://www.genemania.org) is a flexible user-friendly web interface for generating hypotheses about gene function, analyzing gene lists and prioritizing genes for functional assays. Given a query gene list, GeneMANIA extends the list with functionally similar genes that it identifies using available genomics and proteomics data. GeneMANIA also reports weights that indicate the predictive value of each selected data set for the query. GeneMANIA can also be used in a function prediction setting: given a query gene, GeneMANIA finds a small set of genes that are most likely to share function with that gene based on their interactions with it. Enriched Gene Ontology categories among this set can sometimes point to the function of the gene. Seven organisms are currently supported (*Arabidopsis thaliana*, *Caenorhabditis elegans*, *Drosophila melanogaster*, *Mus musculus*, *Homo sapiens*, *Rattus norvegicus* and *Saccharomyces cerevisiae*), and hundreds of data sets have been collected from GEO, BioGRID, IRefIndex and I2D, as well as organism-specific functional genomics data sets. Users can customize their search by selecting specific data sets to query and by uploading their own data sets to analyze.

## INTRODUCTION

The GeneMANIA prediction server (http://www.genemania.org) has been in operation for the past 3 years (1) although prototypes with limited functionality were available from 2008 (2). Details about the server and its prediction accuracy on benchmark data sets are available in the original NAR Web server article (1) and associated publications that describe the label propagation algorithm used by GeneMANIA to find related genes (2–4) and the algorithm used to assign weights to networks (2,5) (see Table 1 for details). The prediction server is complemented by the GeneMANIA Cytoscape app (6), which makes similar functionality available for desktop use in addition to a set of unpublished software tools for power users that can be run from the command line that are described at http://pages.genemania.org/tools/. See Table 2 for a list of additional resources associated with GeneMANIA.

**Table 1.** GeneMANIA network weighting algorithms

Name in ‘advanced options panel’	Validation: *Algorithm name* (citation)
Assigned based on query weights	*Unregularized* (5)
	*GM-2008* (1)
Gene Ontology-based weighting: BP, MF & CC	*SW* (5)
Equal weighting: equal by network	*Uniform* (5)

**Table 2.** GeneMANIA associated resources

Resource	Location	Description
GeneMANIA Cytoscape app	Cytoscape app store	Desktop app with all GeneMANIA functionality as well as current and archival data
	Also see (Montojo *et al.*, 2010)	
Command line tools	http://pages.genemania.org/tools/	Tools for running GeneMANIA in offline, cross-validation mode; also for building and supplementing GeneMANIA data sets
Bulk data download	http://pages.genemania/data/	Description of data formats and links to archived and current data set bulk downloads
GeneMANIA announcements	Google group: genemania-announce	GeneMANIA news and announcements of new releases.
	Twitter: @genemania	
Bug reports and GeneMANIA questions	http://pages.genemania.org/contact/	Form to contact GeneMANIA development team
GeneMANIA data reports	http://www.genemania.org/data_reports/	Compares data releases in terms of numbers of networks, GO annotations and identifiers

In this article, we describe updates to the GeneMANIA prediction server and data sets that have occurred over the past 3 years, provide a brief guide to the various resources associated with the GeneMANIA project and answer some of the frequently asked questions about GeneMANIA received from users. In the interest of space, we assume that the reader has already read our previous NAR Web server article (1).

### GeneMANIA use cases

GeneMANIA answers three main types of biological questions by searching diverse network data types, such as physical, predicted and co-expression interactions. Given a single gene, GeneMANIA will find the most closely connected genes. If the function of the query gene is poorly characterized, identifying interacting genes of known function helps predict the function of the unknown gene (7). Given a query list of genes, GeneMANIA will find more genes like those in the list. In this use, GeneMANIA can be thought of as a ‘gene recommender system’ (4), similar to book (or music or movie) recommender systems, which suggest other books (music or movies) that you might like based on your past selections. If the query list is long enough (see the section on network weighting), GeneMANIA can suggest related genes using the types of connections that strongly connect the query genes. For example, if the query genes are part of a protein complex, GeneMANIA will predict additional members of that complex using primarily physical interactions (which highly connect the query genes); or if the query genes are protein kinases, GeneMANIA will suggest other protein kinases by using the protein domain similarity network. Networks are assigned weights based on how relevant they are to the query genes, and non-relevant networks will receive zero weights and not be shown in the results. Finally, GeneMANIA is a general purpose network search engine that supports selecting networks to search out of hundreds of options to identify a set of connections among query genes (the network weighting method should be set to ‘equal by network’ in this case). This is particularly useful when used in the Cytoscape app to find a network of interactions to process further using Cytoscape’s analysis features.

### GeneMANIA philosophy

#### Don’t get in the way of the data

We have attempted to make our interface and algorithm flexible enough that arbitrary gene networks can be uploaded. Although we depend heavily on annotation and curation efforts of others, we try to avoid doing curation or make choices for the user ourselves. This means that by default, we include all network data that we have for the organism with two exceptions: we currently include only the 20 co-expression data sets that are generally most informative for gene function prediction to speed up queries that use the default settings; also, we do not include functional interaction networks generated using Gene Ontology (GO) annotation because they introduce circularity in our calibration and performance measuring algorithms, which currently rely on performing cross-validation with GO annotation. Nonetheless, both of these network types can be added to the query using the advanced options panel.

#### Ease of use

We try to choose default behavior so that casual users can get useful information quickly: only two mouse clicks are needed for a gene search (choose an organism, type or paste in the gene and press GO!). This means we have made certain decisions about gene identifier mapping: we only recognize gene names that have a unique correspondence with a gene, and we do not do auto-organism detection because in most cases, gene names are shared among organisms. Much of the complexity of GeneMANIA is hidden in the ‘advanced options panel’ that sits under the gene input box and can be opened to change the default behavior.

## GENEMANIA UPDATES

Since our last publication in 2010, we have made a number of updates to the organisms and data contained within GeneMANIA, to our prediction algorithm and to our user interface.

### New organisms and data sources

We have recently added support for another model organism, rat (*Rattus norvegicus*), and are developing zebrafish (*Danio rerio*) and *Escherichia coli* as our next two model organisms to add. Our data set for the supported organisms is updated approximately twice a year, although we occasionally perform smaller updates when large interaction studies are published. Our front page provides up-to-date statistics on the number of interactions and networks in our database. We perform comparisons between the current version of our database and previous ones to identify data issues and track data set growth (see Table 2). We are unable to make previous data releases available through the Web site, but we do archive them for download, and they can be accessed through our Cytoscape app (6). Our app has the same functionality as the Web site, and queries in the app should give identical results as Web site queries. Web site users can download a JSON file to store their search parameters by selecting the ‘Save search parameters as JSON’ option in the File menu of the results page. This file can be read into our Cytoscape app to exactly reproduce the saved search.

As of 1st March 2013, we have almost 1500 networks that collectively contain nearly 300 million interactions covering almost 150 000 genes. All of this data are also available for download from the GeneMANIA data archive (http://pages.genemania.org/data/)—also new since 2010. This site stores current and previous data versions (networks and identifiers) in simple text formats in addition to a ‘precombined’ network that integrates multiple individual GeneMANIA networks into a single large network. This ‘precombined’ network can be used like other published functional interaction networks [recent examples include (8–10)], but the link (i.e., interaction) weights have a slightly different meaning (see below). Currently this precombined network consists of the set of default networks, combined using the GO-based Biological Process method (see Table 1).

### Filtering of co-expression networks

In earlier versions of GeneMANIA, we found the co-expression networks added a lot of interactions without adding much predictive power for GO annotation. As such, we restricted the number of co-expression networks included by default to 20. This also reduces the number of network weights (in the default settings), and thereby reduces risks for overfitting these weights for short query lists [see (5) for details]. However, we have recently added an additional filter on co-expression links. Now, every co-expression link has been seen in at least one other network (co-expression or otherwise), otherwise it is filtered out. This change removed ∼50% of our total interactions with limited change in the AUROC of our GO term predictions and a slight increase in area under the precision-recall curve of our predictions (see ALGORITHM AND SOFTWARE EVALUATION for more details).

### Gene Ontology enrichment analysis

**Figure 1.**
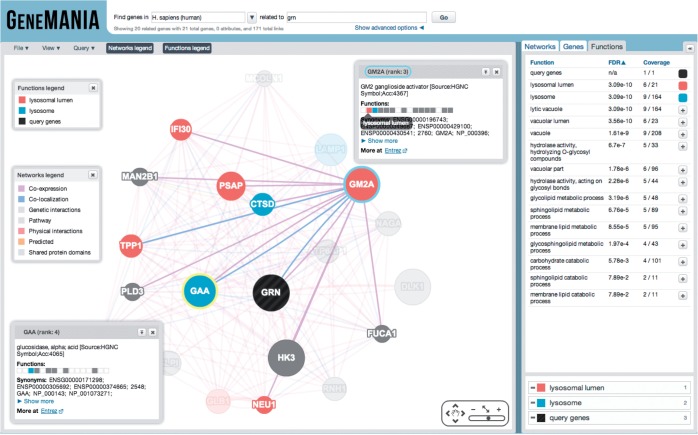
Screenshot of default search for related genes to GRN. Functions tab of the results screen is selected and top two most enriched functions are colored.

For the past 2 years, GeneMANIA has automatically computed Gene Ontology enrichment on the set of genes displayed in the results page (i.e., the query list plus the related genes). The results of this analysis are available through the ‘functions’ tab that also allows coloring of the gene nodes by their annotated function (Figure 1). We also recently introduced the ‘function grid’, which is available in the gene ‘pop-up’ box that appears when you click on a gene. The function grid shows enriched functions for a given gene as a compact grid. Hovering the mouse over a square in the grid shows the enriched term. Highlighting a function term with a color in the Functions tab also colors the corresponding grid square. Opening multiple gene pop-ups simultaneously enables quick comparison of the grids to identify similar functional enrichment patterns among genes.

We test the query list plus the related genes found by GeneMANIA for enrichment of a selection of Gene Ontology term annotations against the background of all genes in the organisms with any Gene Ontology annotation. We use a Benjamini–Hochberg FDR multiple testing correction and report the FDR (also known as the ‘q-value’) associated with each term. We only report GO terms with FDR < 0.05. When doing the enrichment analysis, we only consider GO terms with at least 10 annotations in the organism and no more than 300 to control the size of the multiple testing correction. We currently do not filter the GO terms for redundancy nor do we attempt to merge them into clusters [c.f. (11–13)]. To improve ease of use, we plan to reduce redundancy in this list in the future.

Anecdotally, one can predict gene function for poorly characterized genes by examining the GO enrichment of its related genes (7). For example, GRN is a gene without an annotated sub-cellular localization that was assigned a ‘lysosome’ localization in a recent article (14). The default search for GeneMANIA for GRN shows ‘lysosomal lumen’ and ‘lysosome’ (Figure 1).

### Gene attributes: a new data source and algorithm

**Figure 2.**
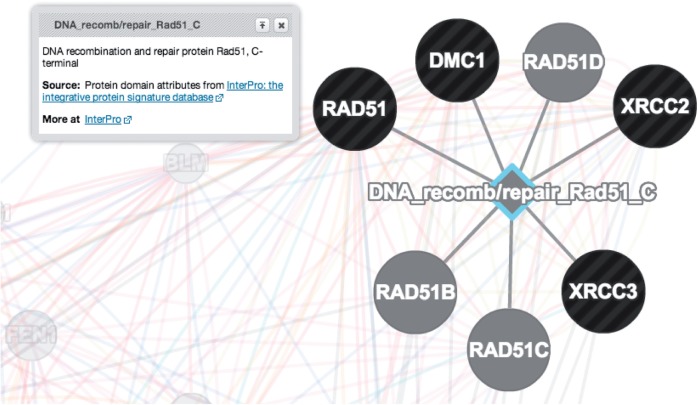
Close-up of attribute node in GeneMANIA results window. Shows attribute node for InterPro domain ‘DNA_recomb/repair_Rad51_C’ linked to genes in the results window that contain that attribute.

We have recently incorporated ‘gene attributes’ as a source of data to use when searching for related genes. These attributes include gene features like ‘the presence of a kinase domain in the protein sequence’ or ‘expression in brain’ and are a valuable source of functional data about genes (15). However, unlike interaction data, these attributes are defined for individual genes. Currently, the GeneMANIA algorithm represents each binary attribute as an interaction network in which all genes with that attribute are linked. In the results page, attributes are represented as a diamond node (see Figure 2 for an example). To accommodate the large increase in the number of networks considered due to this change, we added a small amount of L2-regularization to the linear regressions used by our network weighting algorithms.

We currently only use attributes derived from InterPro domains, although we intend to expand our attribute sets in the near future to include, for example, gene attributes defined by mSigDB (16). Also, attributes are not currently selected by default; users must open the advanced options panel to select them. Because of the large number of possible attributes (and the resulting large number of corresponding networks), GeneMANIA only considers attributes represented in the query list (or query gene) and then only the top N ‘most enriched’ attributes in the query list (where *N* = 20 by default but it can be changed in the advanced options panel). Enrichment is measured by hypergeometric *P*-value.

### PSICQUIC webservice

We have recently shared all GeneMANIA data via a PSICQUIC webservice (17), which allows users to query millions of GeneMANIA functional interactions from their own software and scripts and multiple software that support the standard PSICQUIC molecular interaction query service (such as the Cytoscape PSICQUIC client app). It is accessible through http://webservice.baderlab.org:8180/psi-gm/webservices or through the PSICQUIC Registry: http://www.ebi.ac.uk/Tools/webservices/psicquic/registry/registry?action=STATUS

### GeneMANIA partners

A number of Web sites link to GeneMANIA through our deep-linking interface including BAR (18), SGD (19), Wormbase (20), BioGPS (21). We also link to Entrez, WormBase, FlyBase and SGD from the gene tabs in relevant organisms.

### Other interface changes

We have made a large number of user interface improvements over the past 3 years. Among the most prominent changes are deep linking (see http://pages.genemania.org/help/linking-to-genemania/), gene neighborhood highlighting and network highlighting. The network view has also been updated, and new colors are used to improve contrast. The number of genes and interactions are now displayed with each result, and the advanced options panel is faster. Resetting the layout will set the nodes to their original positions. We have added a query menu option to automatically rerun GeneMANIA on selected nodes. Performing re-query uses the changed query parameters.

Currently, GeneMANIA uses CytoscapeWeb (22), so requires Flash, but we plan to remove this requirement by switching to the new Cytoscape.js HTML5/JavaScript library we have developed (https://github.com/cytoscape/cytoscape.js).

### Saving GeneMANIA results

Users now have more options when saving their GeneMANIA results. The ‘Save’ menu has been changed to the ‘File’ menu. Users can now use a web-app to create a PDF report (the ‘Create Report’ menu item), or save various parts of the network displayed (e.g. ‘save the network as text’ saves the network as tab-delimited text for use with desktop network display tools like Cytoscape), or save the query parameters that they used as a JSON object that can be uploaded to the GeneMANIA Cytoscape app to precisely recreate their query, allowing users to share queries with other users. Pop-up help boxes describe the function of each menu option.

## GENEMANIA FREQUENTLY ASKED QUESTIONS

Users can contact our development team directly though the ‘Contact Us’ page (http://pages.genemania.org/contact/) if they have questions about the interface. The most common questions that we receive are answered in this section. Most of what is described in this section was also true of our original 2010 version of the interface, but we include this here as a convenient resource for users.

### Gene identifiers used by GeneMANIA

We support the following protein-coding gene identifiers, collected from Ensembl and Entrez: Entrez gene, Ensembl gene, official gene symbols, UniProt/SwissProt and RefSeq protein identifiers and unique gene names and synonyms. Any non-unique name or identifier is removed from the system, although official gene symbols are preferentially maintained.

### GeneMANIA automated data build pipeline

Our network data come from six main sources: IRefIndex (23) and BioGRID (24) for physical (i.e., protein) interactions and genetic interactions; Gene Expression Omnibus (GEO) for co-expression networks (25); InterPro, via Ensembl, for protein domains (26); I2D for networks of interologs of physical interactions (27); and from our own manual curation efforts. Gene Ontology annotations are downloaded directly from the Gene Ontology Web site as part of our automated data build, and gene identifiers are retrieved from the Entrez Gene and Ensembl databases. We further process data from IRefIndex, BioGRID and I2D to extract individual interaction networks that are associated with Pubmed IDs. Interaction studies reporting <100 interactions are all consolidated into a single network (e.g. ‘IREF-SMALL-SCALE-STUDIES’). We also consolidate networks by curation source [e.g. ‘IREF-MINT’ (28)], so each interaction is represented in two different networks. In some cases, the same PubMed ID is associated with multiple networks (e.g. those containing interactions detected at stringent and permissive thresholds); in this case, the different networks are represented by appending different letters to the network name. The transformation of mRNA expression profile from GEO to a co-expression network is described later in this article, and the transformation of protein domain profiles to ‘shared domain’ networks has been previously described (2).

### Where to find information about the GeneMANIA data sources

We have two main places to find information about the data sets from the results page. First, this information is available in the Networks panel on the right side of the main network view. Expanding each network category will show you the individual networks used in the network view, and further expanding these will provide information about where the network data comes from e.g. publication/PubMed link and GEO link for co-expression data, protein interaction database for physical interaction data. Hovering the mouse over the network will highlight it in the main network view. Also, clicking on a link will open a pop-up box that tells you which data sources the corresponding interaction was in. Network information is also detailed in the Networks section of the report and also is available when selecting networks in the advanced options panel. Our help page lists a complete list of networks and sources we collect information from (http://pages.genemania.org/help/#Network_data_sources)

### The meaning of the GeneMANIA link weights and node sizes

The link weights reported by GeneMANIA (indicated visually by line thickness) are determined based on the link’s weight in each individual network and the weights assigned each network by our relevance detection algorithms. The link weights in individual networks are determined by a network normalization procedure described below that down-weights links from high degree nodes (i.e. nodes with many neighbors).

We normalize individual networks to make them suitable for GeneMANIA’s label propagation algorithm. In this transformation, each interaction in a physical or genetic network is assigned a link weight of 1/[sqrt(d_i_)sqrt(d_j_)] where d_i_ and d_j_ are the degrees (i.e. number of neighbors) of the two linked genes in the network. In networks where links already have weights (e.g. co-expression networks), the normalized link weights are w_ij_ / [sqrt(wd_i_)sqrt(wd_j_)] where w_ij_ is the initial link weight and wd_i_ and wd_j_ are the ‘weighted degree’ (i.e., the sum of the initial link weights to all neighbors) for the two genes. The post-normalization weighted degree of nodes is approximately one in each network. In addition to being required for the label propagation algorithm, this normalization helps to reduce the impact that the pleiotropy of high degree nodes [see, e.g. (29)] has on functional predictions (30).

The link weights in the ‘composite network’ reported by GeneMANIA in the results page are a weighted sum of the normalized link weights from each network included in the composite times the ‘network weight’ assigned to the network that each link is from.

In the results page, all query genes are given the maximum node size and the size of the nodes for related genes is inversely proportional to the rank of the gene in a list sorted by the gene score assessed by GeneMANIA.

### Network weighting methods

GeneMANIA has multiple methods to combine and weight networks to create the final composite network used to find similar genes. By default, GeneMANIA chooses the network weighting algorithm based on the length of the query list, using the precombined network for queries with less than five genes and the query-weighted method for five or more genes. However, through the advanced options panel, users can choose among seven specific options. These options include ones that depend on patterns of Gene Ontology co-annotation (called ‘Gene Ontology-based weighting’), those that depend only on the gene list (called ‘Assigned based on query genes’) or non-adaptive ones that assign equal weights to each selected network or selected data source. Table 1 shows the GeneMANIA weighting algorithms and the publications where they are described and validated.

The adaptive network weights are determined using a regression-like procedure and roughly represent the added predictive value (when considering all other selected networks) of the links in the network for predicting co-membership in the query list (for ‘Assigned based on query genes’) or co-annotation in Gene Ontology (for ‘Gene Ontology-based weighting’). In general, these network weights roughly represent the usefulness of the network for the prediction task; however, anecdotally, network weights have a small bias toward those that improve the precision of predictions rather than recall. As such, small networks with unique links can sometimes get surprisingly large weights.

### Co-expression networks

GeneMANIA co-expression networks are derived automatically from GEO data series with GSE identifiers. For each data release, we download all GSE series with a minimum number of samples (at least 12 but more for some organisms) that come from a set of GEO platforms that we have pre-defined as measuring mRNA gene expression. For each GSE, we identify the corresponding PubMed ID, which we use to name the network and extract meta-data, and then we compute the Pearson correlation coefficient (*r*) between all pairs of genes. We then sparsify the network by setting to zero any *r* that doesn’t appear in the top 50 highest *r*-values for at least one of the pair of genes. This network then undergoes our normalization procedure described above.

## ALGORITHM AND SOFTWARE VALIDATION

Table 1 provides details on validation for our various network weighting algorithms and our network propagation algorithms. These methods have not changed since our previous NAR Web server article except as described in previous sections and validated here. Filtering unsupported co-expression interactions led to a significant increase (*P* < 10^−^^10^) in the cross-validated area under the precision-recall curve (AUPRC) versus the unfiltered co-expression networks on the task of classifying genes into Gene Ontology biological process terms with between 10 and 100 annotations using GeneMANIA for human, *C.elegans* and yeast (using GeneMANIA data release from 3 March 2011). These were the only species we tested. On the same benchmark but testing only in human and using an internal data build (between the 21 December 2011 and 19 July 2012 releases), adding up to 100 attributes and a small amount of L2-regularization to the network weight regression led to a significant increase (*P* < 10^−^^10^) in AUPRC compared with using just the default network selections in human (median non-zero change in AUPRC was an increase of 0.0078). In terms of software validation, we continue to use unit and functional tests, a modern bug tracking system (Trac) and test GeneMANIA on all major browsers (although we have dropped support for Internet Explorer 7). Beta versions of our next release are posted for external testing at http://beta.genemania.org, and we have a mechanism for user feedback (See Table 2).

## COMPARISONS OF GENEMANIA AND SIMILAR TOOLS

There are a number of tools that offer similar functionality as GeneMANIA and that are available on the web or through Cytoscape. These tools include: STRING (9), N-browse (31), IMP (32), FunctionalNet (33) and FunCoup (34). Each system draws from slightly different data sources and makes different decisions about how to score interactions, and we encourage readers to also evaluate these systems. There are also systems that provide access to a set of pre-defined gene function predictions made on the basis of network and gene attribute data (35).

There are three major differences between GeneMANIA and the majority of these other tools. The first difference is that GeneMANIA’s predictions are made ‘on-the-fly’. Practically, that means that the weights assigned each network change based on what networks the user selects or uploads into GeneMANIA. Another major difference is in the meaning of the link weights. In other systems, the displayed link weights can be interpreted as estimated probabilities that the linked genes ‘functionally interact’, in other words, that they share at least one annotated function. GeneMANIA link weights cannot be interpreted this way, as described in previous sections, though higher weights generally mean higher confidence. A final difference with some servers is that GeneMANIA uses a label propagation algorithm to select related genes [see (30) for the history of this methodology] rather than just considering genes that directly interact with the query list. As such, GeneMANIA considers direct and indirect paths between genes and the query list. Although direct interactions receive more weight than indirect ones, tight clustered groups of genes will often appear together as related genes by GeneMANIA owing to their larger number of indirect links.

## CONCLUSIONS AND FUTURE DIRECTIONS

GeneMANIA remains an open-source project and our code is available on request (though migration to GitHub is planned). Teaching materials are also available on request. Future plans include support for generating instances of GeneMANIA for new organisms, addition of more gene attributes (e.g. phenotype and disease associations, tissue and embryonic stage expression, miRNA target predictions, as well as DNA- and RNA-binding protein targets) and providing web services to enable automated access to the GeneMANIA Web server from third party tools.
